# Clinical and Pathological Characteristics of Autoimmune Hepatitis with Acute Presentation

**DOI:** 10.1155/2018/3513206

**Published:** 2018-03-18

**Authors:** Yi Shen, Changli Lu, Ruoting Men, Jianping Liu, Tinghong Ye, Li Yang

**Affiliations:** ^1^Department of Gastroenterology & Hepatology, West China Hospital, Sichuan University, Chengdu, Sichuan 610041, China; ^2^Department of Pathology, West China Hospital, Sichuan University, Chengdu, Sichuan 610041, China

## Abstract

*Aim. *To study the differences between acute presentation-autoimmune hepatitis (A-AIH) and chronic autoimmune hepatitis (C-AIH).* Methods.* Through long-term follow-up, 80 patients were included in our study by using the revised international autoimmune hepatitis group (IAIHG) score and were divided into acute and chronic groups for comparison.* Results.* No significant difference was found in the gender, age, IAIHG score (pretreatment/posttreatment), definite diagnosis rate, extrahepatic autoimmune disease, onset time, or treatment before biopsy between the acute and chronic groups. In terms of clinical symptoms, A-AIH patients were more prone to jaundice, anorexia, yellow urine, and detesting oil than C-AIH patients, but melena only occurred in chronic group (*P* < 0.05). The acute group exhibited more severe injury upon histological evaluation, with lobular inflammation and bile duct injury, especially central necrosis of the lobule, more pronounced in this group (*P* < 0.05).* Conclusion.* A-AIH had manifestations of acute hepatitis and presented cholestasis. Serum indicators could preliminarily distinguish A-AIH and C-AIH. Histologically, the primary manifestation of A-AIH was lobular inflammation, which was usually accompanied by lobular central necrosis. For the diagnosis of A-AIH, more attention should be paid to long-term follow-up. This study was registered at ClinicalTrials.gov (identifier: NCT02994537).

## 1. Introduction

Typical autoimmune hepatitis (AIH) is a chronic and nonspecific inflammatory disease that is primarily characterized by the presence of autoantibodies, elevated alanine aminotransferase (ALT), aspartate transaminase (AST), and immunoglobulin G (IgG) levels, interface inflammation, lymphoplasmacytic infiltration, and hepatocyte rosetting [1–3]. The common criterion for diagnosing AIH is the international autoimmune hepatitis group (IAIHG) score, but acute presentation-AIH (A-AIH) probably lacks the typical presentations described in this score. Moreover, prominent lobular inflammation and sometimes even obvious central necrosis of the lobule may be present [4–7], which restricts the use of the IAIHG score for A-AIH. Various studies have reported different definitions of A-AIH. Most of these studies have divided the disease into acute-onset AIH, acute exacerbation of chronic AIH (C-AIH), and acute-severe AIH [4, 8–12]. In clinical works, 25%–40% of AIH cases are the acute form [1, 4, 13, 14], so an early and accurate diagnosis of A-AIH to improve the prognosis is an important research goal. Therefore, our study included patients with suspected AIH. Ultimately, 80 patients were included in our study after long-term follow-up. And the patients were divided into acute and chronic groups according to their serum examination results. Then, we analysed the differences between A-AIH and C-AIH. Based on these results, physicians may be able to more easily distinguish A-AIH to improve early diagnoses and increase the timeliness of treatment.

## 2. Materials and Methods

### 2.1. Inclusion Criteria

**Figure 1:**
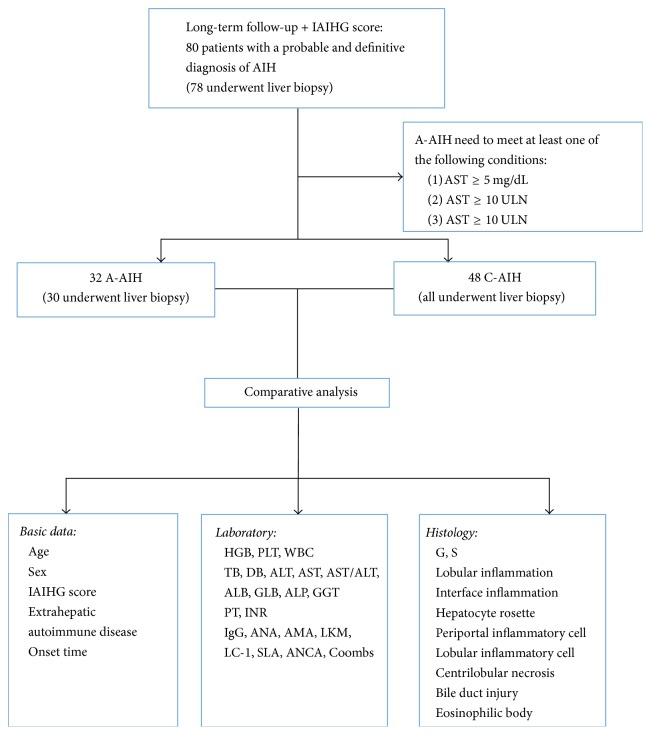
Flow chart. The figure shows the entire process and the contents of the study. IAIHG: international autoimmune hepatitis group; AIH: autoimmune hepatitis; A-AIH: acute presentation-autoimmune hepatitis; TB: total bilirubin; ALT: alanine aminotransferase; AST: aspartate transaminase; C-AIH: chronic autoimmune hepatitis; HGB: haemoglobin; PLT: platelet; WBC: white blood cell; DB: direct bilirubin; ALB: albumin; GLB: globulin; ALP: alkaline phosphatase; GGT: gamma-glutamyl transferase; PT: prothrombin time; INR: international normalized ratio; IgG: immunoglobulin G; ANA: anti-nuclear antibody; AMA: anti-mitochondrial antibody; LKM: liver kidney microsomal; LC: liver cytosol; SLA: soluble liver antigen; ANCA: anti-neutrophil cytoplasmic antibody; Coombs: Coombs test; G: inflammation grade; S: fibrosis stage.

This study enrolled patients who visited the West China Hospital of Sichuan University from October 2013 to November 2016 with suspected AIH. Through long-term follow-up [the median follow-up time for all patients was 14 (11.00, 20.25) months], we ultimately included 80 patients with a probable and definitive diagnosis of AIH based on the 1999 revised IAIHG score (a score ≥10 indicates probable AIH before treatment and a score ≥16 indicates definite AIH before treatment) [15, 16]. Of these patients, 78 patients underwent a liver biopsy as a diagnostic examination, but 2 patients refused to do it. Then, the 80 patients were divided into the A-AIH group and the C-AIH group. For inclusion in the A-AIH group, the patient needed to meet at least one of the following conditions: (1) total bilirubin (TB) ≥ 5 mg/dL, (2) ALT ≥ X10 upper limits of normal (ULN), or (3) AST ≥ X10 ULN. [8–12, 17]. The onset time was defined as the time from the first instance of liver dysfunction to the time of liver biopsy (or the time of admission for patients who did not have a liver biopsy) [4]. Patient information was acquired from the West China Hospital electronic medical record system and by telephone (see Figure 1).

### 2.2. Laboratory Examination

Fasting blood samples were collected from the patients, and routine blood, biochemical, and coagulation function examinations were implemented within 3 days before or after the liver biopsy (or implemented at the time of admission). Other blood test results acquired within 1 month before or after the biopsy were considered acceptable [4]. The laboratory indices included the white blood cell (WBC) and platelet (PLT) counts, haemoglobin (HGB), TB, direct bilirubin (DB), ALT, AST, AST/ALT, albumin (ALB), and globulin (GLB) levels, prothrombin time (PT), international normalized ratio (INR), IgG concentration, the anti-nuclear (ANA), anti-mitochondrial (AMA), anti-liver kidney microsomal (LKM), anti-liver cytosol (LC), anti-soluble liver antigen (SLA), and anti-neutrophil cytoplasmic (ANCA) antibodies, and the Coombs test (Coombs). The laboratory tests were performed by the Department of Laboratory Medicine of West China Hospital, which was certified by the College of American Pathologists (CAP).

### 2.3. Pathological Examination

Liver samples were acquired by ultrasound-guided percutaneous needle biopsy of the liver. The tissues were fixed in 10% formaldehyde (Kelong, China), embedded in paraffin, and used for haematoxylin and eosin (H&E) staining, immunohistochemical staining for CK7 (ZSGB-BIO, China) and CD138 (Maxim, China), and Masson trichrome staining to examine the histological characteristics. Finally, two pathologists (CL and JL) from the Pathology Department of West China Hospital (certified by CAP) interpreted the samples. The Scheuer score system was used to classify the inflammation grade (G0–4) and fibrosis stage (S0–4) of the liver tissues [7, 18]. The pathologists assigned 0 to 4 points for different levels of pathological presentations as follows [4]: (1) lobular inflammation (0–4: no inflammation, piecemeal necrosis, fusion necrosis, multiacinar necrosis, and massive necrosis); (2) interface inflammation (0–3: no, mild, moderate, and severe); (3) hepatocyte rosetting (0 and 1: no and yes); (4) cellular infiltration of the portal area: (0–2: no, few, and many); (5) bile duct injury (0–2: no, bile duct proliferation or vacuolar degeneration, and bile duct deficiency); (6) centrilobular inflammation or necrosis (0 and 1: no and yes); (7) cellular infiltration of the lobule (0–2: no, few, and many); and (8) eosinophilic bodies (0 and 1: no and yes).

### 2.4. Statistical Analysis

Continuous variables that conformed to the normal distribution were expressed by mean ± SD, while continuous variables which did not conform to the normal distribution were expressed using the median and interquartile range. Categorical variables were expressed as numbers (percentages) (see Tables 1–5). Differences between groups were compared using Student's *t*-test, the Mann–Whitney *U* test, and the Chi-square test (*P* < 0.05 was considered significant). Receiver operating characteristic (ROC) curves were used to acquire the cut-off values of valuable parameters. All statistical tests were performed using SPSS version 18.0 (SPSS, Chicago, IL, USA).

## 3. Results

### 3.1. Basic Information

Eighty patients were included, of which 32 (40%) patients had A-AIH and 48 (60%) patients had C-AIH. Thirteen (16.25%) were males, and 67 (83.75%) were females, with a male-to-female ratio of 1 : 5.2. No significant difference was found in the gender, age, IAIHG score (pretreatment/posttreatment), definite diagnosis rate, extrahepatic autoimmune disease status, or the composition of treatment and the time of treatment before biopsy (*P* = 0.458, *P* = 0.471, *P* = 0.696/0.727, *P* = 0.923, *P* = 0.732, *P* = 0.078, and *P* = 0.805, resp.) between the patients with acute-presentation and chronic AIH. The onset time of the A-AIH patients was 6.5 (1.19, 27) months, whereas the onset time of the C-AIH patients was 16 (2.5, 54) months (*P* = 0.054) (see Tables 1 and 4). The common clinical symptoms were shown in Table 5. Jaundice, fatigue, and anorexia were the three most common symptoms in all patients. A-AIH patients were more prone to jaundice, anorexia, yellow urine, and detesting oil than C-AIH patients (*P* < 0.001, *P* < 0.001, *P* = 0.004, and *P* = 0.009, resp.), but melena only occurred in chronic group (*P* = 0.038). Other less common symptoms include itching, diarrhea, vomiting, heart fatigue, and shortness of breath.

### 3.2. Laboratory Examination

The HGB, PLT, WBC, AST/ALT, AMA, LKM, LC-1, SLA, ANCA, and Coombs test results showed no significant differences between the two groups. Bilirubin: TB and DB were higher in the acute group (*P* < 0.001 and *P* < 0.001, resp.). Liver enzymes: ALT and AST were higher in the acute group (*P* < 0.001 and *P* < 0.001, resp.), and ALP and GGT were higher than in the chronic group (*P* < 0.001 and *P* = 0.003, resp.). ALB was higher in the chronic group (*P* < 0.001), whereas GLB was higher in the acute group (*P* = 0.001). Moreover, the cut-off value of ALB deduced by ROC curve was 36.9 g/L (see Figure 3). Regarding coagulation function, the PT was longer (*P* < 0.001) and the INR was greater (*P* < 0.001) in the acute group. IgG was higher in the acute group (*P* < 0.001). In the acute group, 28 (87.5%) patients had an ANA titre greater than or equal to 1 : 100 (1 : 100 was equivalent to the international general 1 : 40 titre in this study [8]) compared with only 26 (55.3%) patients in the chronic group, and the positive rate of ANA was significantly different between the two groups (*P* = 0.003) (see Table 2).

### 3.3. Histological Examination

**Figure 2:**
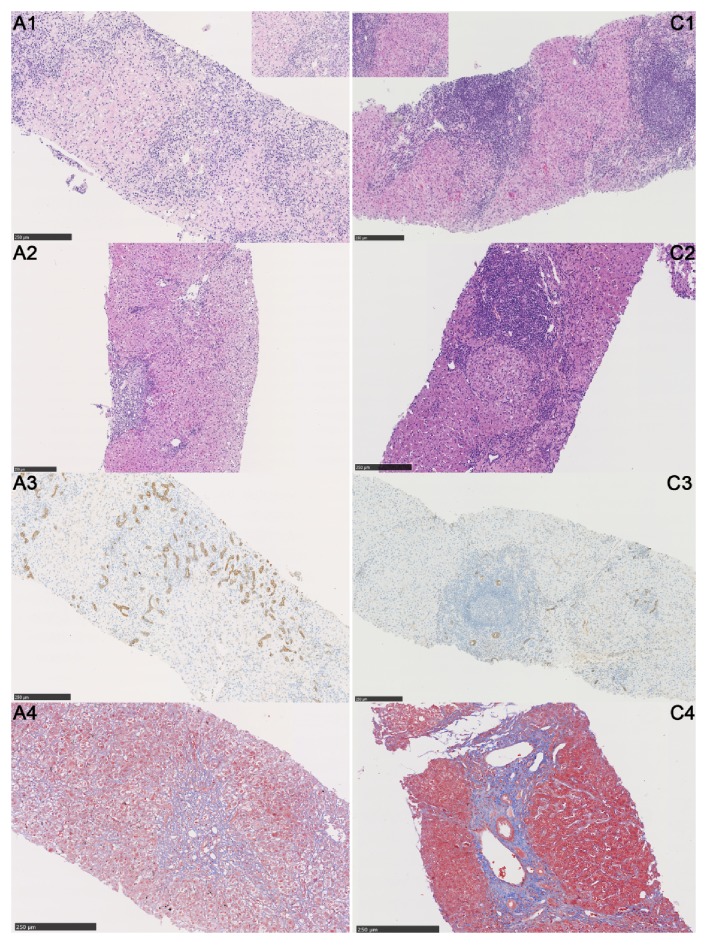
Histological features of A-AIH and C-AIH. A-AIH (A1–4) and C-AIH (C1–4): A1, C1: A1 shows multiacinar and centrilobular necrosis in A-AIH, which is uncommon in C-AIH (H&E, ×200, small pictures emphasized the typical areas in the big pictures); A2, C2: the main manifestation of A-AIH is cellular swelling, ballooning change, and lobular inflammation accompanied by mild interface inflammation; however, C-AIH often has moderate-to-severe interface inflammation caused by lymphoplasmacytic infiltration (H&E ×200); A3, C3: CK7 staining showing regeneration of cholangiocytes due to the multiacinar necrosis of hepatocyte in A-AIH. However, bile duct regeneration was less likely to be found in C-AIH (CK7, ×200); A4, C4: blue staining shows fibrosis in the portal area; fibrous septa were found in the chronic group but not the acute group (Masson trichrome staining, ×200).

**Figure 3:**
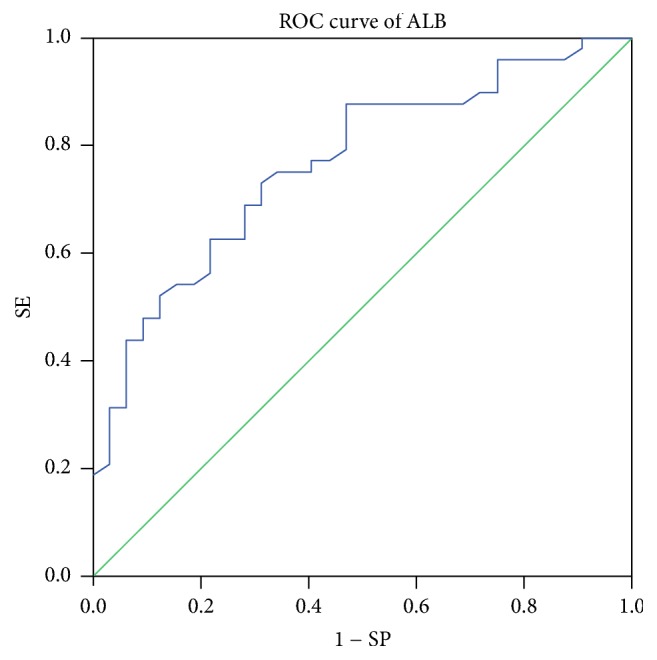
ROC curve of ALB. The area under the receiver operating characteristic (ROC) curve was 0.765 for ALB, 95% confidence interval (CI) was 0.662–0.869, and rates for sensitivity and specificity were 72.9% and 68.7%, respectively.

A-AIH was primarily performed as acute hepatitis in histology. No significant difference in the fibrosis stage (S) was found between the acute and chronic groups. Conversely, the inflammation grade (G) (*P* < 0.001), lobulitis (*P* = 0.001), interface inflammation (*P* = 0.001), rosettes (*P* < 0.001), periportal lymphocytes (*P* < 0.001), plasma cells (*P* < 0.001), neutrophils (*P* = 0.001), monocytes (*P* < 0.001), eosinophils (*P* = 0.002), bile duct injury (*P* < 0.001), centrilobular necrosis (*P* = 0.005), lobular neutrophils (*P* < 0.001), monocytes (*P* < 0.001), eosinophils (*P* = 0.002), and eosinophilic bodies (*P* = 0.05) were all significantly different between the acute and chronic groups (see Table 3 and Figure 2). In the acute group, the patients with onset time less than 3 months and S < 3 were defined as acute-onset AIH, while the rest of the patients were acute exacerbation of chronic AIH (there were 2 patients who had no liver biopsy so they could not be included). After comparison, we found that PLT [206 (165, 276), 106 (70, 161), *P* = 0.018], interface inflammation [no or mild: 100%, 16%, *P* = 0.001], periportal eosinocyte (100%, 48%, *P* = 0.035), and centrilobular necrosis (80%, 24%, *P* = 0.031) between the acute-onset and acute-on-chronic groups were statistically different.

## 4. Discussion

A-AIH can quickly lead to liver decompensation if the diagnosis and treatment are not timely. And A-AIH can be easily confused with other liver dysfunction diseases, leading to misdiagnoses and delayed treatment. Moreover, A-AIH does not usually exhibit the clinical characteristics of typical chronic AIH, which complicates a diagnosis based solely on the existing diagnostic criteria. Therefore, understanding the haematological and histological characteristics of A-AIH is necessary to ensure early diagnosis and treatment.

Previous reports differ in their descriptions of the onset age of A-AIH [10, 13, 19, 20]. In our study, no significant difference was found in the mean age between the two groups; therefore, determining the age of onset of A-AIH requires further study. Moreover, no significant difference was observed in extrahepatic autoimmune disease between the two groups. The two groups of patients (57.1% of A-AIH patients and 44.4% of C-AIH patients) with extrahepatic autoimmune diseases (A-AIH: 4 thyroid disease, 1 systemic lupus erythematosus, and 2 Sjogren's syndrome; C-AIH: 4 thyroid disease, 2 Sjogren's syndrome, 1 Sjogren's syndrome and dermatomyositis, 1 rheumatoid arthritis, and 1 vitiligo) were mainly diagnosed with thyroid disease. The study of Miyake et al. showed that A-AIH concurrent with thyroid disease was often seen in Caucasians, but the inverse was observed in Japanese [10]. Therefore, determining whether autoimmune thyroid disease and A-AIH have the same genetic susceptibility and whether extrahepatic autoimmune diseases in A-AIH patients have ethnic differences requires a larger sample or an international multicentre study for clarification. The onset time of A-AIH has been reported to be 1–3 months in various studies [4, 11]. In our study, no difference was found in the onset time between the two groups (*P* = 0.054). However, the median onset time was significantly lower in the acute group than in the chronic group [6.5 (1.19, 27) and 16 (2.5, 54) months, resp.], possibly because the acute group included patients with acute-onset AIH and acute exacerbation of C-AIH. The results might have been different if we enlarged the sample and divided the A-AIH patients into acute-onset AIH and acute exacerbation of C-AIH groups for separate analyses.

In this study, we only used serum indicators to judge A-AIH without distinguishing acute and chronic histological characteristics; therefore, a portion of the A-AIH patients corresponded to acute exacerbation of C-AIH. However, Abe et al. divided acute and chronic AIH patients using histological characteristics and found that the A-AIH patients also met our blood conditions for A-AIH [9]. This finding indirectly explains the reliability of using blood indicators to distinguish between acute-presentation and chronic AIH. Acute-severe AIH showed markedly abnormal coagulation, and many studies defined AIH with an INR > 1.5 as acute-severe AIH [8, 21–23]. In this study, 6 patients (18.75%) in the acute group but only 1 patient (2.08%) in the chronic group (*P* = 0.015) had an INR > 1.5. Additionally, ALB was lower in the acute group in our study, which also indicated worse A-AIH conditions. Severe AIH patients exhibit severe destruction of the liver tissue, which directly hampers the production of ALB in the liver and explains the difference in ALB between the two groups. Kessler et al. suggested that ALB might play a role in the acute phase response; thus, hypoproteinaemia was a suitable indicator of A-AIH [4]. In our study, the GLB and IgG levels were higher in the acute group than in the chronic group, which was different from the results of many other studies [6, 9, 10, 13, 19]. However, those studies did not include acute exacerbation of C-AIH in their A-AIH groups. Moreover, IgG is not an acute phase protein, and thus A-AIH patients may not have enough time to generate a significant amount of IgG [19, 21]. Miyake et al. showed that A-AIH displayed an IgG concentration <2000 mg/dL; therefore, using the revised IAIHG score to determine A-AIH may correspond to a decreased score and lead to a decreased definite diagnosis of AIH [10]. This finding suggests that the scope of the revised IAIHG score should be reconsidered. In this study, 15 (50%) patients in the A-AIH group had S ≥ 3. Amontree et al. mentioned that the key identification between acute exacerbation of C-AIH and acute-onset AIH might be a higher globulin level [24]. Therefore, GLB and IgG might have been higher in our acute group because more acute exacerbation of C-AIH patients was included in the A-AIH group. They also found that the identification of pathological features between patients with acute exacerbation of C-AIH and acute-onset AIH could include severe liver cell necrosis and fibrosis [24]. Thus, we compared all included patients with S < 3 and S ≥ 3. The results showed that patients with S < 3 had lower GLB [36.1 (31.7, 42.7) g/L and 40.4 (35.7, 47.3) g/L, resp., *P* = 0.012] and IgG [20.6 (16.5, 25.6) g/L and 26.8 (20.7, 32.4) g/L, resp., *P* = 0.019] levels than patients with S ≥ 3. As a result of inflammation, AIH can cause the destruction and proliferation of bile ducts. Duct involvement was more clearly observed in A-AIH than in C-AIH [19]. In this study, 7 (23.33%) patients in the A-AIH group had multiacinar necrosis in the histological analysis, whereas only 4 (8.33%) patients in the chronic group had this condition. When comparing the two groups, we found that both ALP and GGT were higher in the acute group (*P* < 0.001 and *P* = 0.003, resp.). The severe lobulitis in A-AIH may have made the bile duct injury more apparent. This result is consistent with the above studies and indirectly confirms Czaja and Carpenter's opinion that destruction and deficiency of bile ducts cannot rule out the diagnosis of AIH [25]. Onji et al. reported that A-AIH patients had a lower positive rate of ANA than C-AIH patients [6, 10], whereas in our study the positive rate of ANA was higher in the A-AIH patients than in the C-AIH patients (87.5% and 55.3%, resp., *P* = 0.042). This result may be due to the reason that patients with acute exacerbation of C-AIH were included in the acute group. Both the acute and chronic groups in our study included AMA (+) patients (15.6% and 2.1%, resp.). The liver functions of all of the patients were improved even when they only used the standard immunosuppressive treatment without ursodeoxycholic acid (UDCA), and their ALP and GGT levels were decreased or even returned to normal levels. Therefore, abnormal values of the above blood indicators are still likely to be due to the bile duct injury caused by inflammation in A-AIH. Leung et al. found that the AMA appearance may be caused by the oxidative stress induced by liver injury [26].

A number of studies have reported that A-AIH exhibits more severe histological characteristics than C-AIH [4, 12], and the results of our study have confirmed these reports. Many studies have speculated that centrilobular injury may be a manifestation of early AIH, and some studies have reported centrilobular necrosis in 53%–100% of A-AIH cases [4, 6, 7, 10–12, 21, 27, 28]. Hofer et al. reported that centrilobular necrosis suggested a possibility of acute-onset AIH as high as 87% [5]. We also observed that centrilobular necrosis was more obvious in the acute group than in the chronic group. Ten (33.3%) patients in our A-AIH group had centrilobular necrosis, whereas only four (8.33%) patients in the chronic group had this condition (*P* = 0.005). Eleven (78.6%) of the 14 patients had S < 3. Sequential liver biopsies may reveal that A-AIH with centrilobular necrosis gradually appears as a typical AIH with manifestation, thereby assisting with the diagnosis [29]. Therefore, adding centrilobular necrosis to the IAIHG diagnostic criteria should be considered to increase the diagnosis rate of A-AIH by pathologists. Our study indicated that bile duct injury was more likely to occur in A-AIH than in C-AIH. Czaja and Carpenter also reported destructive and nondestructive cholangitis and the disappearance of bile ducts in AIH [25]; thus, a liver biopsy after immunosuppressive treatment may help identify whether the disease is AIH [19, 27]. Amontree et al. reported that fibrosis and cirrhosis could be observed in A-AIH [24]. In our study, the fibrosis stage revealed no significant differences between the acute and chronic groups (*P* = 0.099), with 50% and 31.25% of patients in the acute and chronic groups, respectively, corresponding to S ≥ 3 (*P* = 0.098). Because AIH can suddenly be exacerbated after insidious onset, determining whether acute AIH itself can appear as obvious fibrosis is difficult.

This study has several limitations. For example, the sample size of the A-AIH group was not sufficiently large. Additionally, the results of A-AIH were affected by the C-AIH. In clinical work, we have found that AIH concurrent with viral hepatitis and drug-induced AIH are not uncommon. Therefore, in this study, we also examined viral hepatitis markers in the patients and tracked their medication histories. Through long-term follow-up, we ruled out the possibility of drug-induced liver injury. Finally, patients whose IAIHG scores conformed to the possible diagnosis of AIH were included in this study, but for some patients we could not exclude an effect caused by viral hepatitis.

In conclusion, A-AIH is not uncommon and may not have features of typical AIH, and it is characterized by significantly elevated bilirubin and transaminase. Therefore, serum indicators can preliminarily distinguish A-AIH and C-AIH. Histologically, A-AIH is mainly manifested as acute lobular inflammation with clear centrilobular necrosis, and bile duct injury is not uncommon. Moreover, A-AIH may be difficult to diagnose based only on the IAIHG score. More attention should be paid to long-term follow-up, and new diagnostic criteria for A-AIH should be developed.

## Figures and Tables

**Table 1:** Comparison of basic pieces of information between A-AIH and C-AIH patients.

	Acute *n* = 32 (40.0%)	Chronic *n* = 48 (60.0%)	*P*
Gender (M : F)	1 : 7	1 : 4.3	0.458
Age (years)	52 ± 12	54 ± 13	0.471
Pretreatment IAIHG score	13 (12, 17)	14 (12, 16)	0.696
Posttreatment IAIHG score	14 (13, 20)	15 (12, 18)	0.727
Definite diagnosis (%)	11 (34.4%)	16 (33.3%)	0.923
Extrahepatic autoimmune disease	7 (21.9%)	9 (18.8%)	0.732
Onset time (months)	6.5 (1.19, 27)	16 (2.5, 54)	0.054

**Table 2:** Comparison of laboratory data between A-AIH and C-AIH patients.

	Acute *n* = 32 (40.0%)	Chronic *n* = 48 (60.0%)	*P*
HGB (g/L)	116 ± 21	118 ± 26	0.687
PLT (10^9^/L)	112 (76, 169)	121 (76, 160)	0.757
WBC (10^9^/L)	5.24 (3.51, 6.43)	4.50 (3.72, 5.69)	0.556
TB (*µ*mol/L)	93 (48.1, 198.9)	17.8 (13.5, 24.3)	<0.001^a^
DB (*µ*mol/L)	83.2 (30.3, 157.4)	6.7 (4.7, 11.3)	<0.001^a^
ALT (IU/L)	330 (149, 452)	42 (22, 97)	<0.001^a^
AST (IU/L)	394 (208, 557)	45 (34, 87)	<0.001^a^
AST/ALT	1.21 (0.98, 1.74)	1.13 (0.94, 1.65)	0.694
ALB (g/L)	34.3 ± 5.4	40.1 ± 5.8	<0.001^a^
GLB (g/L)	42.7 (35.8, 47)	35.8 (31.4, 41.3)	0.001^a^
ALP (IU/L)	144 (125, 173)	98 (80, 146)	<0.001^a^
GGT (IU/L)	128 (63, 225)	58 (27, 110)	0.003^a^
PT (s)	14.7 (13.2, 16.3)	12.8 (11.9, 13.4)	<0.001^a^
INR	1.27 (1.12, 1.39)	1.09 (1.01, 1.13)	<0.001^a^
IgG (g/L)	30.8 (21.5, 33.2)	19.3 (16, 24.7)	<0.001^a^

ANA			
<1 : 100	4 (12.5%)	21 (44.7%)	0.003^a^
≥1 : 100	28 (87.5%)	26 (55.3%)	

AMA			
−	27 (84.4%)	43 (97.7%)	0.322
+	5 (15.6%)	1 (2.3%)	

LKM			
−	32 (100%)	43 (97.7%)	1.000
+	0	1 (2.3%)	

LC-1			
−	32 (100%)	43 (97.7%)	1.000
+	0	0	
±	0	1 (2.3%)	

SLA			
−	31 (96.9%)	41 (93.2%)	1.000
+	1 (3.1%)	3 (6.8%)	

ANCA			
−	14 (77.8%)	19 (79.2%)	0.178
+	2 (11.1%)	0	
±	2 (11.1%)	5 (20.8%)	

Coombs			
−	6 (42.9%)	14 (66.7%)	0.091
+	8 (57.1%)	6 (28.5%)	
±	0	1 (4.8%)	

^a^ *P* < 0.05. AIH: autoimmune hepatitis; IAIHG: international autoimmune hepatitis group; HGB: haemoglobin; PLT: platelet; WBC: white blood cell; TB: total bilirubin; DB: direct bilirubin; ALT: alanine aminotransferase; AST: aspartate transaminase; ALB: albumin; GLB: globulin; ALP: alkaline phosphatase; GGT: gamma-glutamyl transferase; PT: prothrombin time; INR: international normalized ratio; IgG: immunoglobulin G; ANA: anti-nuclear antibody; AMA: anti-mitochondrial antibody; LKM: liver kidney microsomal; LC: liver cytosol; SLA: soluble liver antigen; ANCA: anti-neutrophil cytoplasmic antibody; Coombs: Coombs test.

**Table 3:** Comparison of histological features between A-AIH and C-AIH patients.

	Acute *n* = 30	Chronic *n* = 48	*P*
G			
0	0	1 (2.1%)	<0.001^a^
1	1 (3.3%)	12 (25.0%)	
2	2 (6.7%)	17 (35.4%)	
3	21 (70.0%)	18 (37.5%)	
4	6 (20.0%)	0	

S			
0	0	2 (4.2%)	0.135
1	10 (33.3%)	20 (41.7%)	
2	3 (10.0%)	10 (20.8%)	
3	7 (23.3%)	4 (8.3%)	
4	10 (33.3%)	12 (25.0%)	

Lobular inflammation			
0	0	2 (4.2%)	0.002^a^
1	17 (56.7%)	40 (80.3%)	
2	6 (20.0%)	2 (4.2%)	
3	7 (23.3%)	4 (8.3%)	

Interface inflammation			
0	4 (13.3%)	13 (27.1%)	0.001^a^
1	5 (16.7%)	21 (43.8%)	
2	14 (46.7%)	11 (22.9%)	
3	7 (23.3%)	3 (6.3%)	

Hepatocyte rosettes			
No	2 (6.7%)	21 (43.8%)	<0.001^a^
Yes	28 (93.3%)	27 (56.3%)	

Periportal lymphocyte			
0	0	0	<0.001^a^
1	1 (3.3%)	19 (39.6%)	
2	29 (96.7%)	29 (60.4%)	

Periportal plasmocyte			
0	1 (3.3%)	17 (35.4%)	<0.001^a^
1	15 (50.0%)	27 (56.3%)	
2	14 (46.7%)	4 (8.3%)	

Periportal neutrophils			
0	8 (26.7%)	28 (58.3%)	0.002^a^
1	12 (40.0%)	16 (33.3%)	
2	10 (33.3%)	4 (8.3%)	

Periportal monocyte			
0	0	0	<0.001^a^
1	1 (3.3%)	19 (39.6%)	
2	29 (96.7%)	29 (60.4%)	

Periportal eosinocyte			
0	13 (43.3%)	36 (75.0%)	0.005^a^
1	17 (56.7%)	12 (25.0%)	
2	0	0	

Bile duct injury			
0	5 (16.7%)	31 (64.6%)	<0.001^a^
1	21 (70.0%)	17 (35.4%)	
2	4 (13.3%)	0	

Centrilobular necrosis			
No	20 (66.7%)	44 (91.7%)	0.005^a^
Yes	10 (33.3%)	4 (8.3%)	

Lobular neutrophils			
0	8 (26.7%)	40 (83.3%)	<0.001^a^
1	10 (33.3%)	5 (10.4%)	
2	12 (40.0%)	3 (6.3%)	

Lobular monocyte			
0	8 (26.7%)	39 (81.3%)	<0.001^a^
1	11 (36.7%)	7 (14.6%)	
2	11 (36.7%)	2 (4.2%)	

Lobular eosinocyte			
0	19 (63.3%)	44 (91.7%)	0.002^a^
1	10 (33.3%)	4 (8.3%)	
2	1 (3.3%)	0	

Eosinophilic body			
No	22 (73.3%)	44 (91.7%)	0.05
Yes	8 (26.7%)	4 (8.3%)	

^a^ *P* < 0.05.

**Table 4:** Comparison of treatments between A-AIH and C-AIH before liver biopsy.

	Acute *n* = 32 (40.0%)	Chronic *n* = 48 (60.0%)	*P*
No treatment	1 (3.1%)	6 (12.5%)	0.078
General treatment	21 (65.6%)	31 (64.6%)	
General treatment + immunosuppressants	1 (3.1%)	5 (10.4%)	
General treatment + UDCA	8 (25%)	3 (6.3%)	
General treatment + immunosuppressants + UDCA	1 (3.1%)	3 (6.3%)	

Time of treatment (months)	2 (0.50, 12)	1 (0.33, 12)	0.805

**Table 5:** Comparison of symptoms between A-AIH and C-AIH.

	Acute *n* = 32 (40.0%)	Chronic *n* = 48 (60.0%)	*P*
Jaundice	21 (65.6%)	12 (25.0%)	<0.001^a^
Fatigue	14 (43.8%)	18 (37.5%)	0.576
Anorexia	18 (56.3%)	8 (16.7%)	<0.001^a^
Ventosity	10 (31.3%)	13 (27.1%)	0.687
Ascites	9 (28.1%)	13 (27.1%)	0.919
Yellow urine	14 (43.8%)	7 (14.6%)	0.004^a^
Detesting oil	12 (37.5%)	6 (12.5%)	0.009^a^
Abdominal pain	4 (12.5%)	10 (20.8%)	0.337
Nausea	6 (18.8%)	5 (10.4%)	0.333
Weight loss	6 (18.8%)	4 (8.3%)	0.187
Dizziness	2 (6.3%)	8 (16.7%)	0.301
Lower limb swelling	2 (6.3%)	6 (12.5%)	0.466
Arthralgia	1 (3.1%)	6 (12.5%)	0.233
Melena	0	7 (14.6%)	0.038^a^
Hematemesis	0	5 (10.4%)	0.08

^a^ *P* < 0.05.
